# Oxidative Stress in Neurodegenerative Diseases

**DOI:** 10.3390/antiox11030504

**Published:** 2022-03-05

**Authors:** Andrii Domanskyi, Rosanna Parlato

**Affiliations:** 1 Institute of Biotechnology, HiLIFE, University of Helsinki, 00014 Helsinki, Finland; 2 Department of Neurology, Division of Neurodegenerative Disorders, Medical Faculty Mannheim, University of Heidelberg, Theodor-Kutzer-Ufer 1-3, 68167 Mannheim, Germany

Oxidative stress is typically reported in neurodegenerative diseases. Although the mechanisms underlying this condition are not completely understood, the brain appears particularly susceptible to oxidative stress due to its high oxygen consumption and to its vulnerable antioxidant defense. Oxidative stress may directly or indirectly predispose neurons to death as a consequence of mitochondrial dysfunction, altered proteostasis, physiological neurotransmitter metabolism, inflammation, or the deregulation of antioxidant pathways. Increased levels of reactive oxygen species (ROS) and reactive nitrogen species (RNS) and impaired antioxidant defense mechanisms are frequently observed in most neurodegenerative diseases, such as Alzheimer’s, Parkinson’s, Huntington’s disease, and amyotrophic lateral sclerosis (ALS). Several studies based on pharmacological and genetic animal and cellular models of neurodegenerative diseases indicate a causative role for ROS and RNS in the damage to proteins, lipids, and nucleic acids, compromising cellular functions and activating cell death pathways. Genetic mutations in the components of the antioxidant defense pathways also show the importance of oxidative stress vulnerability in neurodegenerative processes.

This Special Issue represents an up-to-date collection of seven original research articles and two reviews displaying the multiple ways oxidative stress may contribute to neurodegeneration in various neuronal and disease contexts in clinical and preclinical settings.

Despite several setbacks in past clinical trials, the quest to identify antioxidant compounds that have potent neuroprotective activity continues, which is reflected by several articles in this Special Issue. The Special Issue opens up with Liou C. et al.’s original research article showing that the cholinesterase inhibitor therapy, such as memantine, known to be beneficial in Alzheimer‘s disease (AD) patients, reduces the oxidative thiobarbituric acid relative substances (TBARS) in AD blood samples in comparison to non-AD controls [1]. Importantly, this study addresses the question of whether individuals carrying the epsilon 4 allele of the apolipoprotein E (APOE4) gene show high levels of TBARS and low levels of anti-oxidant thiols in association with lower mitochondrial DNA copy numbers. In summary, this article explores new avenues to develop reliable disease progression biomarkers, which are critically needed for neurodegenerative diseases.

Nguyen C.D. and Lee G. reported encouraging results in the amelioration of oxidative stress and learning and memory deficits induced by the expression of the beta-amyloid Aβ_25–35_ fragments. This study shows the neuroprotective mechanism of the honeybee venom, Melittin, in a mouse hippocampal cell line and in an in vivo mouse model based on the Aβ_25–35_ ICV injection for the first time. Treatment with Melittin regulates the nuclear translocation of nuclear factor erythroid 2-like 2 (Nrf2) and the production of the antioxidant enzyme heme oxygenase-1 (HO-1). Moreover, Melittin administration could ameliorate the learning and memory performance of the Aβ_25–35_-treated mice. By activating the Tropomyosin-related kinase receptor B (TrkB)/cAMP Response Element-Binding (CREB)/Brain-derived neurotrophic factor (BDNF), Melittin also regulates synaptic function [2]. Ongoing studies aim at reducing the side effects of Melittin and implementing its dosage and delivery in patients.

Wang W. et al. report interesting and original new observations regarding the cross-talk between the excitatory neurotransmitter glutamate and dopamine oxidation [3]. This study identifies several mechanisms by which glutamate might regulate dopamine oxidation, a process that is recognized to have a toxic impact in Parkinson’s disease (PD). These glutamate-mediated mechanisms include, among others, the inhibition of dopamine autoxidation, the copper-mediated oxidation of catecholamines, as well as the formation of toxic quinoproteins. Although this scenario remains to be demonstrated in vivo and at a physiological concentration of dopamine, the authors identified a particular molar glutamate/dopamine ratio (lower than 200) as inhibiting dopamine oxidation.

Another original research article by Mustafa R. et al. focused on understanding the role of the primary cilium, a specialized protrusion of the plasma membrane, for the maintenance of dopaminergic neurons under basal conditions and upon induction of mitochondrial and oxidative stress in a pharmacological mouse model of PD [4]. By generating a mutant mouse line that lacked primary cilia in dopaminergic neurons, this study shows that primary cilia may play a role in the auto-inhibitory action of dopamine on dopaminergic neuron spontaneous activity and that dopaminergic neurons are in part protected by the neurotoxic effect of PD-toxin 1-methyl-4-phenyl-1,2,3,6-tetrahydropyridin (MPTP), an inhibitor of the mitochondrial complex I activity. Future studies should address the impact of primary cilia loss for dopamine-dependent motor and cognitive behavior.

With a different perspective, the study by Bobadilla M., Hernandez C. et al. explores the impact of oral administration of grape juice supplemented with red grape polyphenols on the brain of stressed mice [5]. The molecular readout of the beneficial effects of this newly developed drink was the increased expression of genes related to protection against oxidative stress, such as Nrf2, as well as the increased activity of antioxidant enzymes, such as superoxide dismutase or catalase.

Chemotherapy drug 5-fluorouracil (5-FU) is known to increase intracellular reactive oxygen species (ROS), interfere with cellular antioxidant defense system, and inhibit neurogenesis in rat hippocampus after intravenous injections. In their study, Suwannakot K. et al. investigated the ability of melatonin to reduce these adverse effects of 5-FU [6]. Melatonin administration starting nine days before the first 5-FU injection restored normal levels of doublecortin, Nrf2, and BDNF in 5-FU-treated animals. Moreover, melatonin normalized the levels of selected antioxidant enzymes (catalase, glutathione peroxidase, and superoxide dismutase) and reduced lipid peroxidation (assessed by measuring the levels of malondialdehyde) in the hippocampus and prefrontal cortex. Furthermore, melatonin administration reduced the number of p21-positive cells in the subgranular zone of the dentate gyrus, suggesting its effect on preventing 5-FU-induced cell cycle arrest. Interestingly, these effects were observed only in combination with 5-FU treatment but not after the administration of melatonin alone. Unfortunately, while speculating about potential effects on synaptic plasticity and memory, the authors did not address the behavioral phenotype of the animals in the study. Considering the availability of melatonin as a dietary supplement and its ability to cross the blood–brain barrier, it would be very interesting to investigate if orally administered melatonin would exhibit neurorestorative effects when applied after 5-FU treatment in future studies.

Pro-survival and anti-apoptotic effects of erythropoietin have been actively studied in the past, however, with controversial results. Revisiting the topic, Rey F. et al. studied neuroprotective and neurorestorative activities of erythropoietin in in vitro and in vivo models of Parkinson’s disease [7]. The authors demonstrated erythropoietin efficacy in restoring cell viability and levels of the dopaminergic marker tyrosine hydroxylase (TH) and NURR1 (a nuclear transcription factor that regulates the expression of TH and of the dopamine transporter). Moreover, erythropoietin attenuated mitochondrial dysfunction in 1-methyl-4-phenylpyridinium (MPP^+^)-treated SH-SY5Y cells, a widely used dopaminergic neuron-like cellular model. Importantly, erythropoietin also exhibited neurorestorative activity in MPTP-treated mice, both on the behavioral and molecular levels, being able to restore striatal levels of TH and NURR1 and reduce astrogliosis and microglia activation. Combined with previous data demonstrating the neuroprotective effect of erythropoietin in 6-OHDA-treated mice, another toxin-induced model of PD, there is compelling evidence for its potential efficacy as PD treatment. However, the necessity for intracerebral administration of erythropoietin due to its poor ability to cross the blood–brain barrier greatly limits its therapeutic potential. It remains to be seen if, for example, small molecule mimetics of erythropoietin that are capable of crossing the blood–brain barrier, binding the erythropoietin receptor, and activating downstream signaling pathways could be developed for oral or intravenous delivery.

The role of oxidative stress as a potential driver of neurodegeneration is supported by recent research and is reviewed in two articles of this Special Issue. Sanz-Moreno B. et al. thoroughly review existing evidence on the involvement of oxidative stress in optic neuropathies, characterized by degeneration of retinal ganglion cells and their axons [8]. Increased levels of oxidative stress and alterations in antioxidant defense mechanisms are consistently observed in glaucoma, ischemic optic neuropathy, optic neuritis, and hereditary and environmental toxin-induced optic neuropathies. While the existing data are compelling, it is still insufficient to firmly establish the causative effect of oxidative stress in these conditions. The authors highlight several crucial issues, such as the importance of patient stratification, sufficient population size, consistency of the inclusion criteria and the necessity to consider confounding effects of other diseases, which need to be critically addressed in future studies.

Oxidative stress is inherently linked to the activation of cell death pathways, such as apoptosis and programmed necrosis (necroptosis). Necroptosis is a form of non-apoptotic programmed cell death taking place when apoptosis is blocked. Two members of the receptor-interacting serine/threonine-protein kinase (RIP) family, RIP1 and RIP3, play a key role in executing necroptosis. In their review, Jantas D. and Lason W. carefully describe molecular mechanisms of RIP1-dependent apoptosis and necroptosis and highlight the interplay between these cell death pathways and oxidative stress [9]. Accordingly, several small molecules known to inhibit various stages of necroptosis exhibit neuroprotective effects in cellular models of oxidative stress, excitotoxicity, hypoxia, intracerebral hemorrhage, PD, and Alzheimer’s disease, with some of these results also confirmed in in vivo models. Acknowledging the complexity of interactions between cell death signaling pathways, oxidative stress, and genetic and environmental factors, the authors focus on searching substances with multipotential action and/or combinations of drugs targeting neuronal cell death pathways with antioxidants as a potential strategy to develop disease-modifying treatments for neurodegeneration.

Altogether, the articles included in this Special Issue reflect active and multifaceted scenarios in the field of antioxidant research on neurodegeneration. They provide new models, new potential therapeutical targets aiming at protecting neurons from oxidative stress, and new candidates for developing reliable and sensitive biomarkers. Although the main antioxidant therapies failed in the past, these emerging pathways and mechanisms might help to better understand disease mechanisms triggered by oxidative stress. The field is actively developing and progressing towards identifying clinical candidates, bringing important knowledge to develop disease-modifying strategies and therapies. We hope that this research collection will contribute valuable information that will advance the race to eradicate neurodegenerative diseases.

## References

[1] Liou C.-W., Chen S.-H., Lin T.-K., Tsai M.-H., Chang C.-C. (2021). Oxidative Stress Biomarkers and Mitochondrial DNA Copy Number Associated with *APOE4* Allele and Cholinesterase Inhibitor Therapy in Patients with Alzheimer’s Disease. Antioxidants.

[2] Nguyen C.D., Lee G. (2021). Neuroprotective Activity of Melittin—The Main Component of Bee Venom—Against Oxidative Stress Induced by Aβ_25–35_ in In Vitro and In Vivo Models. Antioxidants.

[3] Wang W., Wu X., Yang C.S., Zhang J. (2021). An Unrecognized Fundamental Relationship between Neurotransmitters: Glutamate Protects against Catecholamine Oxidation. Antioxidants.

[4] Mustafa R., Rawas C., Mannal N., Kreiner G., Spittau B., Kamińska K., Yilmaz R., Pötschke C., Kirsch J., Liss B. (2021). Targeted Ablation of Primary Cilia in Differentiated Dopaminergic Neurons Reduces Striatal Dopamine and Responsiveness to Metabolic Stress. Antioxidants.

[5] Bobadilla M., Hernández C., Ayala M., Alonso I., Iglesias A., García-Sanmartín J., Mirpuri E., Barriobero J., Martínez A. (2021). A Grape Juice Supplemented with Natural Grape Extracts Is Well Accepted by Consumers and Reduces Brain Oxidative Stress. Antioxidants.

[6] Suwannakot K., Sritawan N., Prajit R., Aranarochana A., Sirichoat A., Pannangrong W., Wigmore P., Welbat J. (2021). Melatonin Protects against the Side-Effects of 5-Fluorouracil on Hippocampal Neurogenesis and Ameliorates Antioxidant Activity in an Adult Rat Hippocampus and Prefrontal Cortex. Antioxidants.

[7] Rey F., Ottolenghi S., Giallongo T., Balsari A., Martinelli C., Rey R., Allevi R., Giulio A., Zuccotti G., Mazzucchelli S. (2021). Mitochondrial Metabolism as Target of the Neuroprotective Role of Erythropoietin in Parkinson’s Disease. Antioxidants.

[8] Sanz-Morello B., Ahmadi H., Vohra R., Saruhanian S., Freude K.K., Hamann S., Kolko M. (2021). Oxidative Stress in Optic Neuropathies. Antioxidants.

[9] Jantas D., Lasoń W. (2021). Preclinical Evidence for the Interplay between Oxidative Stress and RIP1-Dependent Cell Death in Neurodegeneration: State of the Art and Possible Therapeutic Implications. Antioxidants.

